# Clinical features and predictors of severity in COVID-19 patients with critical illness in Singapore

**DOI:** 10.1038/s41598-021-81377-3

**Published:** 2021-04-05

**Authors:** Ser Hon Puah, Barnaby Edward Young, Po Ying Chia, Vui Kian Ho, Jiashen Loh, Roshni Sadashiv Gokhale, Seow Yen Tan, Duu Wen Sewa, Shirin Kalimuddin, Chee Keat Tan, Surinder K. M. S. Pada, Matthew Edward Cove, Louis Yi Ann Chai, Purnima Parthasarathy, Benjamin Choon Heng Ho, Jensen Jiansheng Ng, Li Min Ling, John A. Abisheganaden, Vernon J. M. Lee, Cher Heng Tan, Raymond T. P. Lin, Yee Sin Leo, David C. Lye, Tsin Wen Yeo, Singapore 2019 novel coronavirus outbreak research team, Poh Lian Lim, Brenda Sze Peng Ang, Cheng Chuan Lee, Lawrence Soon U. Lee, Oon Tek Ng, Monica Chan, Kalisvar Marimuthu, Shawn Vasoo, Chen Seong Wong, Tau Hong Lee, Sapna Pradip Sadarangani, Ray Junhao Lin, Mucheli Sharavan Sadasiv, Deborah Hee Ling Ng, Chiaw Yee Choy, Glorijoy Shi En Tan, Yu Kit Tan, Sean Wei Xiang Ong, Stephanie Sutjipto, Pei Hua Lee, Jun Yang Tay, Ding Ying, Bo Yan Khoo, Woo Chiao Tay, Gabrielle Ng, Yun Yuan Mah, Wilnard Tan, Sennen Jin Wen Lew, Raymond Kok Choon Fong, Helen May Lin Oh, Jaime Mei Fong Chien, Humaira Shafi, Hau Yiang Cheong, Darren Cheng Han Teo, Thuan Tong Tan, Ban Hock Tan, Jenny Guek Hong Low, Limin Wijaya, Indumathi Venkatachalam, Ying Ying Chua, Benjamin Pei Zhi Cherng, Yvonne Fu Zi Chan, Ghee Chee Phua, Ken Junyang Goh, Jade Xiao Jue Soh, Shuwei Zheng, Pushpalatha Bangalore Lingegowda, Wee Ming Peh, Yi Lin Lee, Jun Yang Ho, April Yu Jie Chia, Li Lin, Say Tat Ooi, Tambyah Paul Anantharajah, Jyoti Somani, Jolene Ee Ling Oon, Gabriel Zherong Yan

**Affiliations:** 1 Tan Tock Seng Hospital, Singapore, Singapore; 2 National Centre for Infectious Diseases, Singapore, Singapore; 3 Sengkang General Hospital, Singapore, Singapore; 4 Changi General Hospital, Singapore, Singapore; 5 Singapore General Hospital, Singapore, Singapore; 6 National University Hospital, Singapore, Singapore; 7 Khoo Teck Puat Hospital, Singapore, Singapore; 8 Ng Teng Fong General Hospital, Singapore, Singapore; 9 Lee Kong Chian School of Medicine, Novena Campus Clinical Sciences Building, 11 Mandalay Rd, Singapore, 308232 Singapore; 10 Duke-NUS Medical School, Singapore, Singapore; 11 Yong Loo Lin School of Medicine, Singapore, Singapore; 12 Ministry of Health, Singapore, Singapore; 13 Saw Swee Hock School of Public Health, Singapore, Singapore; 14 Menzies School of Health Research, Charles Darwin University, Darwin, Australia

**Keywords:** Respiratory distress syndrome, Viral infection

## Abstract

We aim to describe a case series of critically and non-critically ill COVID-19 patients in Singapore. This was a multicentered prospective study with clinical and laboratory details. Details for fifty uncomplicated COVID-19 patients and ten who required mechanical ventilation were collected. We compared clinical features between the groups, assessed predictors of intubation, and described ventilatory management in ICU patients. Ventilated patients were significantly older, reported more dyspnea, had elevated C-reactive protein and lactate dehydrogenase. A multivariable logistic regression model identified respiratory rate (aOR 2.83, 95% CI 1.24–6.47) and neutrophil count (aOR 2.39, 95% CI 1.34–4.26) on admission as independent predictors of intubation with area under receiver operating characteristic curve of 0.928 (95% CI 0.828–0.979). Median APACHE II score was 19 (IQR 17–22) and PaO2/FiO2 ratio before intubation was 104 (IQR 89–129). Median peak FiO2 was 0.75 (IQR 0.6–1.0), positive end-expiratory pressure 12 (IQR 10–14) and plateau pressure 22 (IQR 18–26) in the first 24 h of ventilation. Median duration of ventilation was 6.5 days (IQR 5.5–13). There were no fatalities. Most COVID-19 patients in Singapore who required mechanical ventilation because of ARDS were extubated with no mortality.

## Introduction

In December 2019, a cluster of severe pneumonia patients linked to an animal wholesale market was reported in Wuhan, Hubei Province, China^1,2^. Subsequently the responsible pathogen was identified as the novel zoonotic severe acute respiratory syndrome coronavirus 2 (SARS-CoV-2)^3^. Hitherto in this century, two other zoonotic beta-coronaviruses, SARS-CoV and Middle East Respiratory Syndrome coronavirus (MERS-CoV) have caused outbreaks of severe respiratory disease with estimated mortality rates of 9% and 30% respectively^4^.

Since the initial reports, the number of cases diagnosed with coronavirus disease 2019 (COVID-19) has increased exponentially in China and globally. While the number of reported cases is decreasing in China, the number of cases in other countries is growing at an alarming rate. While approximately 80% of cases will experience mild to moderate disease, single-centre studies from hospitals in Wuhan reported that 16–29% of hospitalized patients developed acute respiratory distress syndrome (ARDS) and 26–32% required intensive care management^1,2,5^. Case fatality rates in these reports ranged between 4.3 and 15%^1,2,5^ A report of 52 critically ill patients from a tertiary referral hospital in Wuhan described ARDS in 67% of patients with a case fatality rate of 62.5%^6^. Currently, there are no reports on clinical details and outcomes of critically ill COVID-19 patients in countries outside China, making it difficult to understand the threat COVID-19 poses in different healthcare settings^7^.

Singapore is a country in Southeast Asia with high levels of travel and economic connectivity with China. During the SARS outbreak in 2003, it was one of the worst affected countries, with 238 patients and 33 deaths, including a significant number of healthcare workers^8^. Similarly, during the current outbreak, Singapore was one of the first countries outside China to diagnose a significant number of SAR-CoV-2 infections and document local transmission^7^.

In this study, we describe the clinical features and treatment outcomes of the first ten COVID-19 patients in Singapore who required mechanical ventilation and compared the clinical, laboratory and radiological features with fifty COVID-19 patients who did not need invasive ventilatory support. We also explored independent predictors for invasive ventilation in these patients. The findings will not only be important for clinical management and triage of infected patients, but will have implications for resource planning across the globe, as countries prepare to deal with the current crisis.

## Materials and methods

### Study design and participants

This was a multicenter case control study of patients with confirmed COVID-19 by SARS-CoV-2 real-time polymerase chain reaction (RT-PCR) using a previously described method^7^. All COVID-19 patients were admitted for treatment and isolation in government hospitals with negative pressure facilities. Airborne and contact precautions were observed and staff attending to patients wore personal protective equipment in accordance with the United States Centers for Disease Control and Prevention guidelines. Collection of de-identified clinical data from infected individuals was approved by the Ministry of Health, Singapore under the Infectious Disease Act with waiver of written informed consent. All methods were carried out in accordance with relevant guidelines and regulations.

### Data collection

We recorded demographic, clinical, laboratory and radiological data for COVID-19 patients using a standardized case report form modified from the International Severe Acute Respiratory and Emerging Infection Consortium^9^. Information collected included epidemiological data (age, gender, ethnicity, exposure to COVID-19 cases, travel history, clinical symptoms and comorbidities), vital signs on admission and transfer to the intensive care unit (ICU), laboratory values on admission and ICU transfer (hemoglobin; white blood cell, neutrophil, lymphocyte and platelet counts; lactate dehydrogenase [LDH], C-reactive protein [CRP], creatinine, arterial blood gas), fraction of inspired oxygen concentration (FiO_2_), radiological findings and treatment (oxygen therapy, antibiotics, oseltamivir, interferon beta-1b, lopinavir-ritonavir and inotropes) for all patients admitted to ICU until 20th February 2020. Data collection was completed on 27th February 2020 for the above, and was censored for patients still in ICU (mortality, days to extubation, and extracorporeal membrane oxygenation) on day of manuscript submission. A non-ICU cohort was selected from the first fifty consecutive patients admitted to participating hospitals who recovered without requiring mechanical ventilation. Two researchers individually reviewed the data forms, and all inconsistent data was clarified with the attending doctors, patients or their families.

The date of disease onset was defined as the day the symptoms were first noted. We defined ARDS following recommendations from WHO and the Berlin criteria^10^. Acute respiratory illness was defined as patients who developed respiratory failure requiring mechanical ventilation without fulfilling the Berlin criteria. Acute kidney injury was identified according to the Kidney Disease: Improving Global Outcomes definition^11^. For ICU patients, the Sequential Organ Failure Assessment (SOFA) and Acute Physiology and Chronic Health Evaluation II (APACHE) scores were recorded on admission.

### Clinical management

Respiratory samples were tested for influenza and other respiratory viruses with a multiplex PCR assay, and urine for pneumococcal and legionella antigen if clinically indicated. Serial nasopharyngeal swabs for SARS-CoV-2 RT-PCR were done for all patients. Supportive therapy including supplemental oxygen was provided according to the degree of hypoxia and the decision for transfer to ICU was made by the attending physician and intensivist. Patients clinically suspected of community-acquired or ventilator-associated pneumonia were administered empiric broad-spectrum antibiotics and oral oseltamivir according to the treating physician’s discretion. Lopinavir-ritonavir (400 mg/100 mg twice daily orally for up to 14 days) and interferon beta-1b (8 million units subcutaneously every other day) was prescribed to selected patients, mainly in the ICU. Corticosteroids were avoided with concern for reported increased mortality in patients with SARS and MERS^12^.

### Outcomes

We compared the demographic, clinical, laboratory and radiological differences on admission between patients who required and did not require invasive mechanical ventilation. The incidence of ARDS, shock, mechanical ventilation, dialysis and mortality were reported.

### Statistical analysis

Continuous variables were expressed as mean (95% confidence interval) or median (interquartile range) depending on distribution, and categorical variables were expressed as frequency and percentage. We compared differences for continuous variables using two-sample *t* test or Wilcoxon rank-sum test depending on the distribution, and χ^2^ test or Fisher’s exact test for categorical variables. To assess the predictive utility of continuous variables for invasive ventilation, the area under the receiver operating characteristic (AUROC) curve and the 95% confidence interval (CI) were calculated.

A multivariable logistic regression model was developed to identify predictors of intubation after exclusion of individuals who were intubated at presentation. Variables were chosen if complete data was available and considered biologically relevant or determined as significantly different between ICU and non-ICU groups on univariate analysis. All selected variables were included in the model and then removed by backward elimination if the p-value was < 0.1. The resulting logistic regression equation was used to estimate the logit(probability) for each individual in the study, and the probability back transformed to generate the receiver operating characteristic (ROC) and the area under the curve (AUC) with 95% CI of the equation. A simplified model was generated using only categorical variables.

Tests were two-sided with significance level set at < 0.05. Analyses were performed using MedCalc Statistical Software version 19.1.7 (MedCalc Software Ltd, Ostend, Belgium) and STATA 13.1 (StataCorp, College Station, Texas, USA).

## Results

### Demographic and clinical data

A total of sixty confirmed COVID-19 cases were included in the study with fifty managed in the general ward and ten who required invasive mechanical ventilation. The mean age was 44 years (95% CI 41–47), 37 (62%) were male and 17 (28%) reported comorbidities (Table 1). The mean symptom duration of symptoms before admission was 5.1 days (95% CI 3.9–6.2) with common complaints being fever (47 [78%]) and cough (46 [77%]).

**Table 1:** Baseline characteristics and laboratory findings of patients infected with SARS-CoV-2 on admission to hospital and ICU.

	All	Non-ICU	ICU	CI for difference in means p-value*
N	60	50	10	
Age, years, mean (95% CI),	44 (41–47)	43 (39–46)	52 (44–59)	0.03
Sex, male, no. (%)	37 (62)	29 (58)	8 (80)	0.29
Chinese ethnicity, no. (%)	52 (87)	44 (88)	8 (80)	0.61
Travel in the 2 weeks before symptoms onset, no. (%)	18 (30)	26 (52)	3 (30)	0.30
Any comorbidity, no. (%)	17 (28)	12 (24)	5 (50)	0.13
**Symptoms**				
Duration of symptoms prior to admission, days, mean (95% CI)	5.1 (3.9–6.2)	4·9 (3.6–6.3)	5.7 (4.1–7.3)	0.63
Fever, no. (%)	47 (78)	37 (74)	10 (100)	0.10
Cough, no. (%)	46 (77)	39 (78)	8 (80)	1.00
Dyspnea, no. (%)	16 (25)	8 (16)	8 (80)	< 0.001
Sore throat or coryza, no. (%)	29 (48)	27 (54)	2 (20)	0.08
Diarrhea, no. (%)	10 (17)	9 (18)	1 (10)	1.00
**Baseline vital signs (range)**				
Temperature, °C	37.7 (37.5–38.0)	37.6 (37.3–37.8)	38.7 (38.1–39.2)	0.001
Heart rate, beats per minute	93 (89–97)	89 (85–93)	111 (99–123)	< 0.001
Respiratory rate, breaths per minute	18.4 (18.0–18.9)	18.0 (17.6–18.4)	20.5 (18.9–22.1)	< 0.001
Systolic blood pressure, mmHg	129 (125–133)	128 (123–132)	136 (121–151)	0.16
Pulse oximeter oxygen saturation, %	97.3 (96.7–98.0)	97.9 (97.5–98.3)	94.4 (91.7–97.1)	< 0.001
**Baseline blood investigations (range)**				
White blood cell, × 10^9^/L	4.9 (4.4–5.4)	4.6 (4.2–5.0)	6.3 (4.3–8.2)	0.01
Lymphocyte, × 10^9^/L	1.2 (1.1–1.4)	1.3 (1.2–1.5)	0.6 (0.4–0.8)	< 0.001
Neutrophil, × 10^9^/L	3.2 (2.7–3.6)	2.7 (2.4–3.0)	5.3 (3.4–7.2)	< 0.001
Neutrophil:lymphocyte ratio	4.0 (2.8–5.3)	2.6 (2.0–3.1)	11.5 (6.4–16.5)	< 0.001
Hemoglobin, g/dL	14.0 (13.6–14.4)	14.1 (13.7–14.5)	13.3 (12.4–14.2)	0.10
Platelet, × 10^9^/L	200 (180–219)	201 (178–223)	195 (150–239)	0.84
Creatinine, µmol/L	69 (64–73)	67 (62–72)	77 (64–90)	0.11
ALT U/L (n = 46)	38 (31–44)	32 (27–37)	59 (38–80)	0.002
CRP, mg/L (n = 47)	52 (35–69)	29 (17–41)	137 (91–182)	< 0.001
LDH, U/L (n = 51)	562 (485–639)	486 (440–532)	919 (601–1237)	< 0.001
LDH:lymphocyte ratio (n = 51)	763 (530–996)	487 (388–585)	2052 (1091–3012)	< 0.001
**Radiology, no. (%)**				
Abnormal chest radiograph	30 (50)	21 (42)	9 (90)	0.01
**Viral shedding mean (95% CI)**				
Duration of viral shedding**	15.4 (13.5–17.3), n = 45	15.1 (13.1–17.1), n = 39	17.8 (12.0–23.6), n = 6	0.34

Only available data were analyzed. N (%) or mean and 95% confidence intervals.

*ICU* intensive care unit, *ALT* alanine transaminase, *CRP* C-reactive protein, *LDH* lactate dehydrogenase.

*Continuous variables compared with t-test, dichotomous with Fisher’s exact. All continuous variables assessed to be approximately normal by Mann–Whitney U.

**From symptom onset to last detectable PCR.

Patients requiring mechanical ventilation were significantly older compared to those that did not (mean 52 years, 95% CI 44–59 versus 43 years, 95% CI 39–46). Dyspnea was significantly more common in those who were intubated (8 [16%] vs. 8 [80%]).

### Vital signs and laboratory data

Temperature, heart rate and respiratory rate of ventilated patients on admission were significantly increased, and oxygen saturation decreased compared with non-ventilated patients (Table 1). Leukocyte and neutrophil counts were significantly higher and lymphocyte count lower in ICU versus non-ICU patients resulting in an increased neutrophil/lymphocyte ratio. Alanine aminotransferase, CRP and LDH were significantly higher in ICU versus non-ICU patients. Significantly more patients requiring mechanical ventilation had abnormal chest X-ray on admission compared with those that did not (9[90%] vs. 21[42%]). Duration of viral shedding in respiratory samples was similar between the two groups.

### Predictors of mechanical ventilation

**Figure 1:**
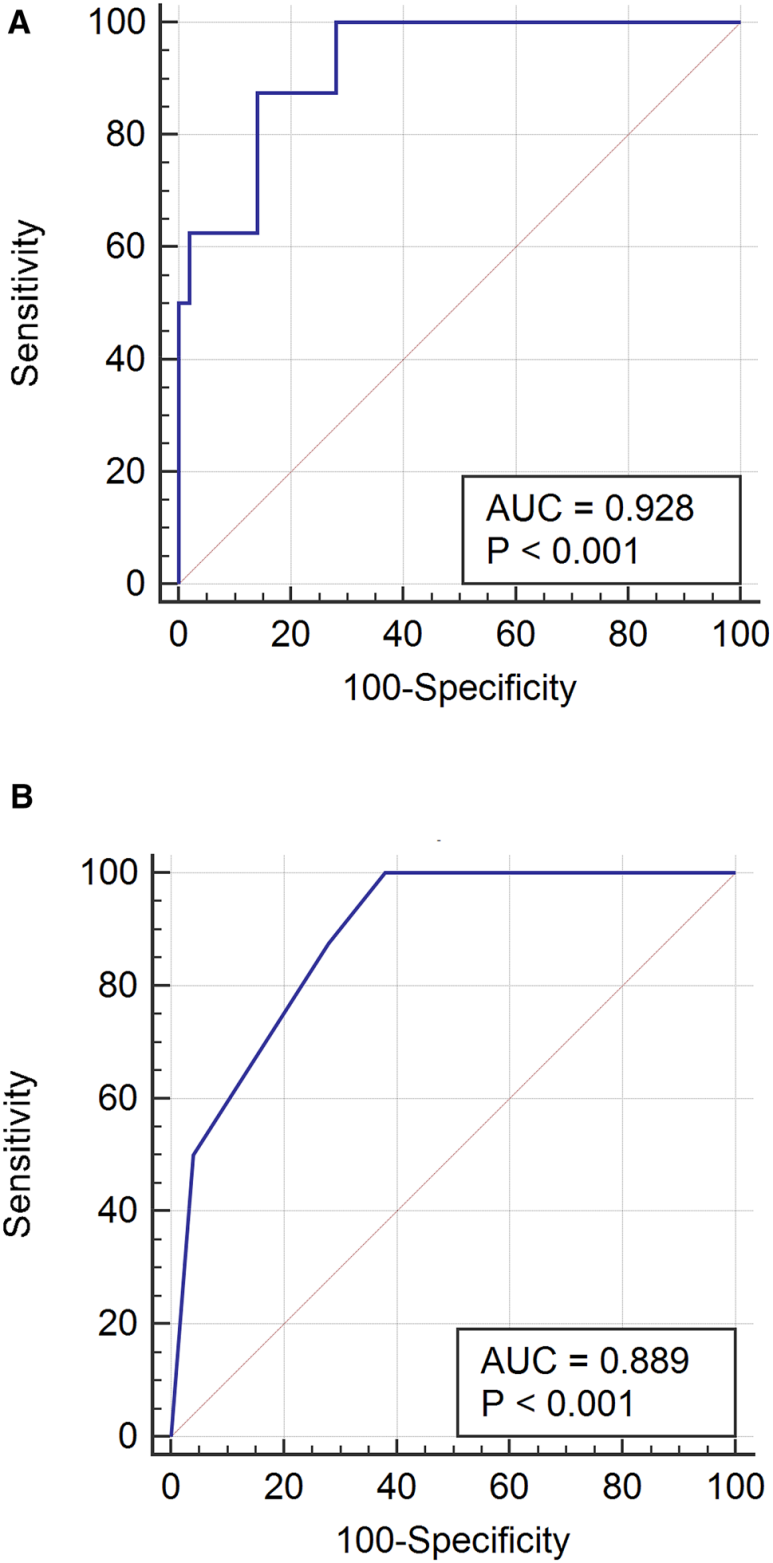
Summary of univariate odds ratios and multivariable logistic regression model. (**A**) AUC for full model 0.928 (95% CI 0.828–0.979, p < 0.0001). (**B**) Simplified model includes the following categorical variables as risk factors for intubation: neutrophil count > 4 × 10^9^ L and respiratory rate > 18. AUROC for this model is 0.889 (95% CI 0.779–0.956, p = 0.0002). AUROC: Area under receiver operating characteristic.

Two patients were intubated at the emergency department and excluded from analysis; the remaining eight cases and 50 controls were included. Area under the receiver operating characteristic (AUROC) curves of baseline neutrophil and lymphocyte counts, neutrophil:lymphocyte ratio, CRP and LDH indicated that CRP > 68.7 mg/L offered the best discriminatory power for predicting intubation (AUROC 0.932, 95% CI 0.816–0.986). The neutrophil:lymphocyte ratio AUROC of 0.885 (95% CI 0.774–0.954) added no additional value to lymphocyte count alone of 0.883 (95% CI 0.771–0.952) (Supplementary Table E1). The multivariable logistic regression retained neutrophil count and respiratory rate as predictors of intubation (Table 2, Fig. 1). A full multivariate model including both as continuous variables generated an AUROC of 0.928 (95% CI 0.828–0.979, p < 0.0001) (Fig. 1A). A simplified model using the categorical variables neutrophil count > 4 × 10^9^/L and respiratory rate > 18 breaths per minute had an AUROC of 0.889 (95% CI 0.779–0.956, p = 0.0002) (Fig. 1B). These criteria offered excellent negative predictive value, as neutrophilia and tachypnea were absent in 31/50 (62%) of patients not requiring intubation while 0/8 (0%) intubated patients met these criteria. Conversely, 4/8 requiring intubation had neutrophilia and tachypnoea, which were present in only 2/50 (4%) who did not.

**Table 2:** Baseline characteristics and laboratory results of mechanically ventilated COVID-19 patients on day of ICU admission.

Patient number	Age (years)	Gender	Co-morbidities	Onset of Symptoms to Intubation (days)	Time from hospital admission to Intubation (days)	Respiratory rate (/min)*	Heart rate (/min)*	Mean arterial pressure (mmHg)*	White blood cells (× 10^9^/L)	Neutrophil count (× 10^9^/L)	Lymphocyte count (× 10^9^/L)	Lactate dehydrogenase (U/L)	Creatinine (µmol/L)	C-reactive protein (mg/L)
1	47	Female	Nil	7	6	22	92	65	6.8	5.84	0.52	650	55	190.5
2	52	Male	Diabetes mellitus, fatty liver, obesity	9	1	24	92	66	5.1	4.39	0.5	646	81	56
3	39	Male	Nil	6	1	46	102	62	8.2	6.91	0.76	1908	73	202.2
4	71	Male	Diabetes mellitus	9	3	45	113	68	6.2	5.23	0.61	632	62	248.7
5	62	Male	Gastroesophageal reflux	8	0	32	133	65	9.6	8.70	0.49	1460	93	112.9
6	36	Male	Nil	6	0	26	145	69	2.3	2.02	0.25	396	76	291.1
7	39	Female	Nil	9	4	34	114	58	5.91	NA	0.34	NA	67	178
8	54	Male	Hypertension, hyperlipidaemia	8	1	33	126	62	6.87	NA	0.55	402	110	115
9	64	Male	Nil	9	3	30	92	62	11.6	10.56	NA	896	78	140
10	53	Male	Diabetes mellitus without chronic complications, obesity	10	1	33	162	61	10.4	NA	0.4	NA	67	199.6

*NA* not available.

*Highest readings for respiratory rate and heart rate while lowest for mean arterial pressure in the first 24 h of intensive care unit admission.

### Details of mechanically ventilated patients

On 25 February 2020, twelve patients required mechanical ventilation out of a total of 91 COVID-19 patients in Singapore for a risk of 13.1%. Due to a short duration of follow-up time, two ICU patients were not included in this study.

For the ten included patients, median time from symptom onset to hospital admission was 8.5 days (IQR 7–9) and median time to intubation after admission was 1 day (range, 0.8–3.3), with two intubated in the emergency department (Supplementary Table E2). The median APACHE II score was 19 (IQR 17–22) and SOFA score 8 (IQR 5–10) (Supplementary Table E3). Nine patients met ARDS criteria on transfer to ICU and one had acute respiratory injury without other complications. The median PaO2/FiO2 ratio before intubation was 104 (IQR 89–129) increasing to 168 (IQR 134–217) post-intubation. During the first 24 h of ventilatory support, the median peak FiO2 was 0.75 (IQR 0.6–1.0), positive end-expiratory pressure (PEEP) 12 (IQR 10–14) and plateau pressure was 22 (IQR 18–26). Five (50%) patients required paralysis, and two (20%) prone positioning to maintain oxygenation. No patient required extracorporeal membrane oxygenation. One patient developed acute kidney injury requiring renal replacement therapy, three patients developed shock requiring inotropes for a median of 1 days (IQR 1–5), one had cardiac injury with elevated troponin, and four had nosocomial infections (one with *Candida krusei* fungemia, three with ventilator-associated pneumonia with endotracheal aspirate cultures positive for *Serratia marcescens, Enterobacter aerogenes, Pseudomonas aeruginosa and Elizabethkingia species*) (Supplementary Table E4). There was also one complication each of upper limb deep vein thrombosis and gastrointestinal bleeding.

All mechanically ventilated patients received lopinavir-ritonavir and three received interferon beta-1b. Three patients were treated with lopinavir-ritonavir and interferon beta 1b (median APACHE II 23, IQR 20–25); two were still intubated for 24 and 30 days. Seven were treated with lopinavir-ritonavir alone (median APACHE II 18, IQR 16–20) with median duration of intubation of 6 days (IQR 5–7). Four patients received at least one dose of oseltamivir and all were treated with antibiotics for a median of 7 days (IQR 2–11) (Supplementary Table E4). No patient received corticosteroids. Respiratory viral multiplex PCR, urinary antigen for pneumococcal and Legionella and other bacterial cultures were negative.

At the time of writing, eight patients were extubated and two remained on ventilatory support with no fatalities. Two patients were re-intubated, with one able to be extubated on the second attempt while the second remained intubated. Of the eight extubated patients, the median duration of intubation was 6.5 days (IQR 5.5–13); four patients were discharged and four remained in the general ward. Of the two patients still intubated, the duration of intubation for the first is currently 30 days. For the remaining patient, he was extubated after eleven days but was reintubated after 6 days with a new diagnosis of nosocomial pneumonia. There was no significant difference in age, APACHE II and SOFA scores, and PaO2/FiO2 ratio between patients who were extubated and those still intubated.

### Radiological findings in critically ill patients

**Figure 2:**
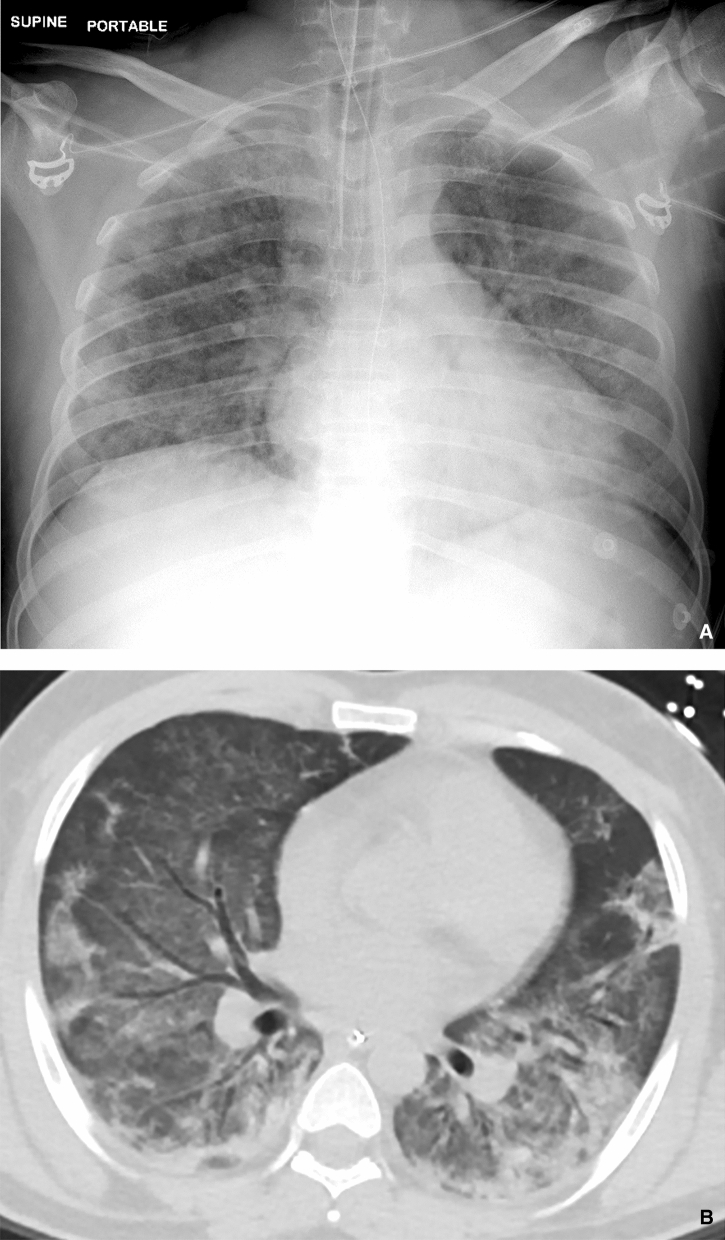
(**a**) Portable supine CXR shows intubated patient with diffuse mixed patchy ground glass opacities with consolidation predominantly in the peripheries. There is no zonal predilection, hilar adenopathy or associated pleural effusion. (**b**) Axial contrast enhanced CT (CECT) image across the lower zones of the lungs confirms CXR features. There are multifocal areas of consolidation in the peripheries, notably in the dependent regions of the lower lobes. Ground glass opacities are more diffusely distributed. Air bronchograms are salient throughout the lower lobes. There is notable absence of pleural effusions or adenopathy in the mediastinum (not shown).

**Figure 3:**
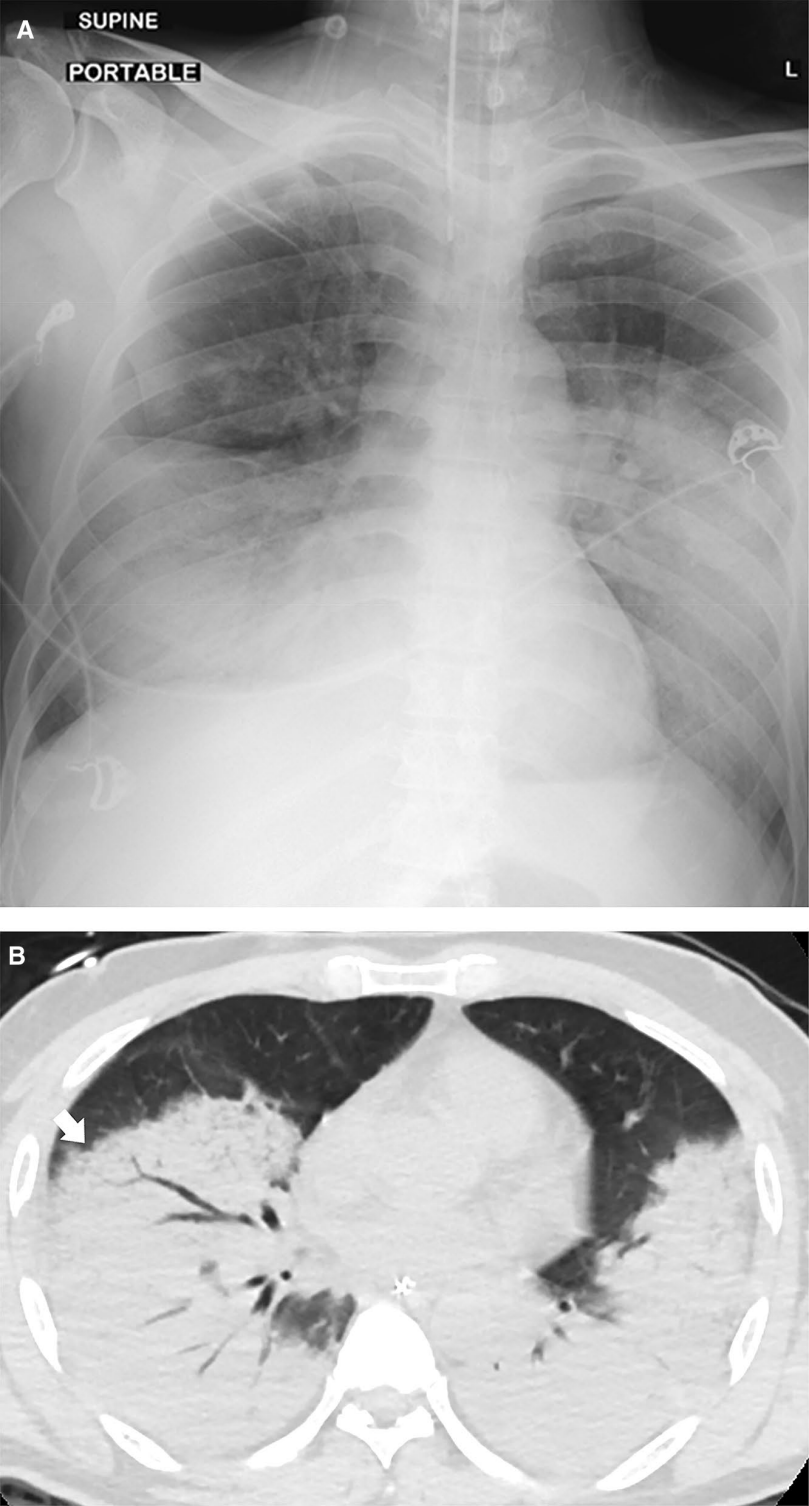
(**a**) Portable supine CXR shows intubated patient with dense consolidation in bilateral middle and lower zones. There is relative sparing of the upper zones and costophrenic recesses. There is no associated pleural effusion. (**b**) Axial CECT image across the middle zone of the lungs shows consolidation with air bronchograms throughout the affected lobes, particularly bilateral lower lobes. The more anterior segments show some degree of organizing consolidation (arrow). As with other cases in our series, there is absence of pleural effusions or adenopathy in the mediastinum (not shown). This pattern of confluent consolidation was a less typical observation.

**Figure 4:**
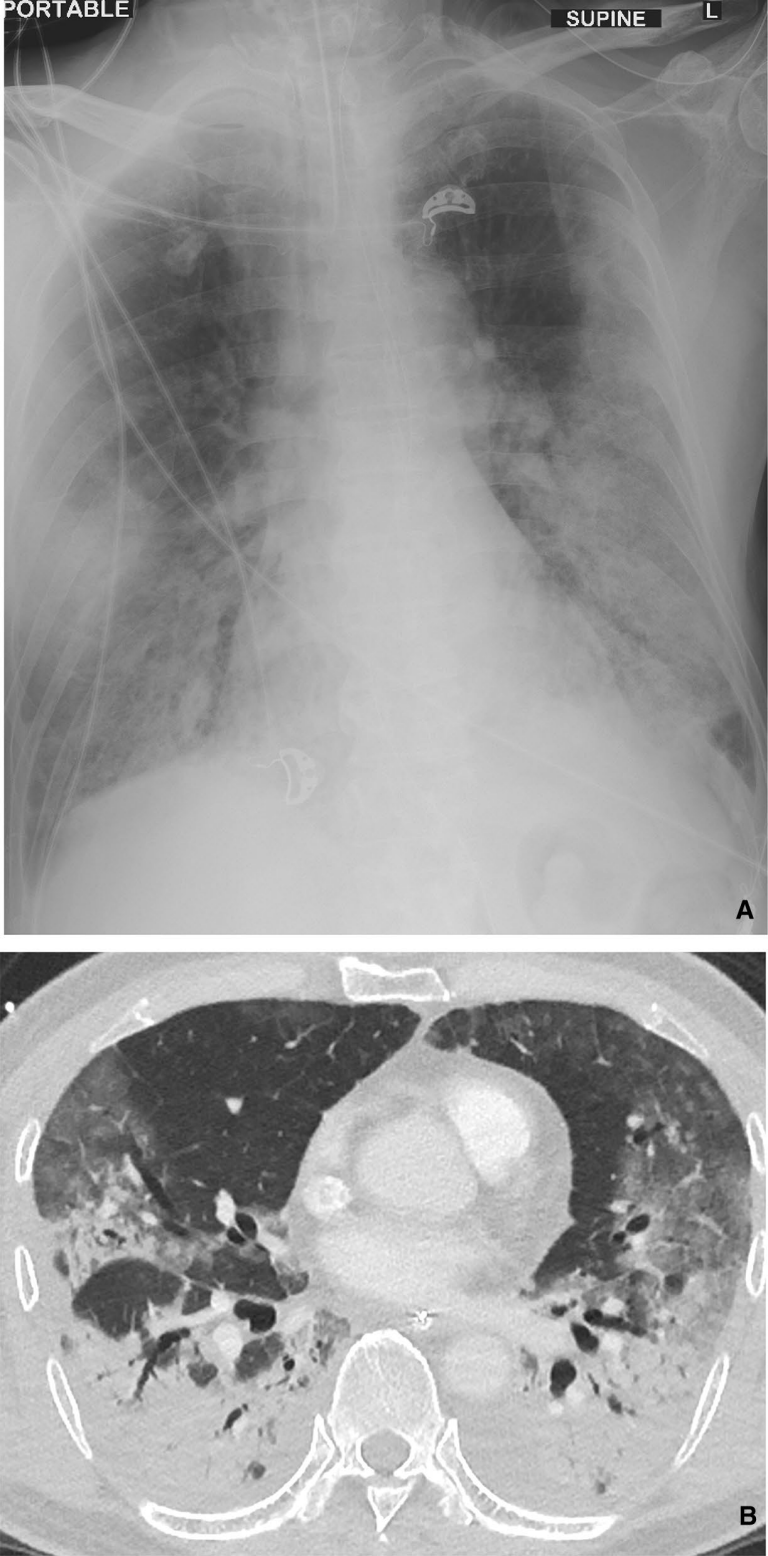
(**a**) Portable supine CXR shows intubated patient with mixed ground glass opacification and consolidation in the peripheries of the middle and lower zones. There is relative sparing of the central and upper zones. There is no associated pleural effusion. (**b**) Axial high resolution reconstructed CECT image across the middle zone of the lungs shows extensive bilateral lung disease with relative sparing of the anterior lobar segments. Consolidation is most notable in the superior segments of the lower lobes with smaller foci in the posterior segment of the right upper lobe. There are ground glass opacities in the lateral regions, distinct from the non-diseased medial portions of the right upper and left lingular lobe.

Figures 2, 3 and 4 showed the radiographic images of three patients and their corresponding computed tomography (CT) images as a representation of imaging findings in the cohort patients who were intubated. Generally, all patients had extensive lung involvement with predominance in the lower zones on radiographs. CT depicted diffuse ground-glass opacities in the affected regions with varying degrees of organizing and confluent consolidation that appeared to parallel disease severity.

## Discussion

In this case control study of 60 COVID-19 patients, fifty were managed in the general ward and ten in intensive care. The median time of symptom onset to admission was 4 days and to ventilatory support 8.5 days with ARDS being the major complication in nine of the ten ICU patients. At presentation to hospital, older age, the presence of dyspnoea, increased temperature, pulse and respiratory rates, higher leukocyte and neutrophil counts, increased ALT, CRP and LDH, abnormal chest radiograph, decreased oxygen saturation and lymphocyte counts were associated with an increased risk of invasive ventilation.

In assessing predictors for intubation, elevated CRP and decreased lymphocyte count were found to be reliable indicators with AUROC of 0.932 and 0.883 respectively. Additionally, we found a combined model of neutrophil count > 4 × 10^9^/L and respiratory rate > 18 breaths per minute was a reliable predictor for invasive mechanical ventilation. These predictors performed best at identifying individuals who did not require ventilation and may be useful as part of the rapid triage of patients who could be managed as outpatient in the event of a large outbreak if healthcare resources become stretched.

Two single-centre retrospective reports from Wuhan, China described the clinical features and outcomes in 36 and 52 critically ill COVID-19 patients who required ICU care^5,6^. The proportion with ARDS was 61% and 67% with case fatality rates of 26·1% and 62·5% respectively. Evidence from Canada, Singapore and Hong Kong of critically ill SARS patients and a Saudi Arabian study of MERS patients recorded mortality rates of 43%, 38%, 26% and 58% respectively^13–16^. Currently, there have been no deaths in the first ten mechanically ventilated COVID-19 patients in Singapore although two remain intubated. The APACHE II, SOFA score, PaO2/FiO2 ratio and age range in our patients were comparable to the Wuhan ICU reports.

The main complication in our patients was acute hypoxemic respiratory failure with the imaging patterns consistent with a report of severely ill patients in China^17^. The development of ARDS is consistent with a report on the pathological findings in a fatal case of COVID-19 with pulmonary complications^18^. Most patients received supplemental oxygen via nasal cannula, Venturi masks and masks with reservoir bags prior to intubation although two patients were intubated on admission. The PaO2/FiO2 ratio improved marginally after intubation and application of positive pressure. High PEEP and FiO2 were required to maintain adequate oxygenation in all patients during the first 24 h. Despite that, plateau and driving pressures for all patients remained low, suggesting the lungs remained compliant and hypoxia may result from shunt mechanism. The hypoxemia was responsive to PEEP with no complications even at higher PEEP settings. No patients required extracorporeal membrane oxygenation.

Compared with reports from Wuhan, the proportion of acute kidney injury (AKI) appeared lower in our patients (10%) while the incidence of cardiac injury and shock (30%) was similar. In Wuhan, AKI was reported as a complication in 8,·3–30% of patients in two reports while 30.6% had shock and 22% developed cardiac injury^6^.

Hitherto, Singapore has reported 16 deaths from COVID-19, out of which 8 demised in the ICU. The proportion of Singapore patients with severe COVID-19 appeared similar to a report using national data from China at around 13–14%^19^. However another recent Chinese study found that only 5% required ICU admission^20^. Compared with case fatality among critically ill COVID-19 patients from Wuhan^5,6^, our case fatality rate is low. This may be due to Singapore’s relatively low number of critically ill patients compared with Wuhan, where healthcare facilities were overwhelmed. The Ministry of Health, Singapore introduced nationwide policies aimed at containing local spread and preventing an explosive outbreak. Close contacts of confirmed cases were identified and quarantined, while all confirmed cases isolated in hospitals.

In a mathematical model of COVID-19 transmission, highly effective contact tracing and case isolation was found to be adequate to control the overall number of cases and a new outbreak within 3 months^21^. Due to the stringent screening processes and compulsory admission of confirmed COVID-19 patients, we were able to monitor for early deterioration and admit to ICU for elective intubation. The association between healthcare resource availability and outcome may account for significant differences in COVID-19 mortality between different geographical regions around the world, but also highlights the importance of containing an outbreak to ensure healthcare resources can cope effectively^22^.

Our study has several limitations. First, only 60 patients with confirmed COVID-19 infections were included. While the variables identified as predictors of invasive mechanical ventilation are clinically plausible, the data is likely to suffer from over-fitting given these small numbers. Validation in a larger cohort will be necessary to determine the optimal variable selection. Second, we only analysed patients requiring invasive mechanical ventilation and did not include a small number of patients who had high oxygen requirements without ventilatory support. The strengths of this study are that it was multicenter and the data prospectively collected. Our report is the first outside of China with detailed description on critically ill COVID-19 patients and the independent predictors for invasive mechanical ventilation.

In summary, ARDS was the major complication in critically ill COVID-19 patients. Given adequate resources and manpower, it is possible to intervene in a timely manner to reduce complications and possibly mortality.

## Supplementary Information

Supplementary Information 1.

Supplementary Information 2.
