# Supplementary material for: Clinical features and predictors of severity in COVID-19 patients with critical illness in Singapore
Source: Sci Rep. 2021 Apr 5;11:7477. doi: 10.1038/s41598-021-81377-3 (PMC8021583; doi:10.1038/s41598-021-81377-3)
Supplement: Supplementary file 1 — Supplementary Information 1. [file 41598_2021_81377_MOESM1_ESM.doc]

Author list:

Ser Hon Puah, MBBS^1^, Barnaby Edward Young, MBBchir^1,2,9^, Po Ying Chia, MBBS^1,2,9^, Vui Kian Ho, MBBS^3^, Jiashen Loh, MBBS^3^, Roshni Sadashiv Gokhale, MBBS^4^, Seow Yen Tan, MBBS^5^, Duu Wen Sewa, MBBS^5^, Shirin Kalimuddin, MBBS^5,10^, Chee Keat Tan, MD^8^, Surinder KMS Pada, MBBS^8,11^, Matthew Edward Cove, MBChB^6,11^, Louis Yi Ann Chai, MBBS^6,11^, Purnima Parthasarathy, MBBS^7^, Benjamin Choon Heng Ho, MBBS^1^, Jensen Jiansheng Ng, MBBS^1^, Li Min Ling, MBBS^1,2,9^, John A Abisheganaden, MBBS^1,9^, Vernon JM Lee, MBBS^12,13^, Cher Heng Tan, MBBS^1,9^, Raymond TP Lin, MBBS^2,11^, Yee Sin Leo, MBBS^1,2,9,11,13^, David C Lye, MBBS^1,2,9,11^, Tsin Wen Yeo, MBBS^1,2,9,14^ on behalf of Singapore 2019 novel coronavirus outbreak research team.

^1^Tan Tock Seng Hospital, Singapore,^2^National Centre for Infectious Diseases, Singapore, ^3^Sengkang General Hospital, Singapore, ^4^Changi General Hospital, Singapore, ^5^Singapore General Hospital, Singapore, ^6^National University Hospital, Singapore, ^7^Khoo Teck Puat Hospital, Singapore, ^8^Ng Teng Fong General Hospital, Singapore, ^9^Lee Kong Chian School of Medicine, Singapore, ^10^Duke-NUS Medical School, Singapore, ^11^Yong Loo Lin School of Medicine, Singapore, ^12^Ministry of Health, Singapore, ^13^Saw Swee Hock School of Public Health, Singapore, ^14^Menzies School of Health Research, Charles Darwin University, Darwin, Northern Territory, Australia

Team authors

| **Name** | **Highest Degree** | **Affiliation / Institution** |
| --- | --- | --- |
| Poh Lian Lim | MD | National Centre for Infectious Diseases, Singapore |
| Brenda Sze Peng Ang | MPH | National Centre for Infectious Diseases, Singapore |
| Cheng Chuan Lee | MMed | National Centre for Infectious Diseases, Singapore |
| Lawrence Soon U Lee | PhD | National Centre for Infectious Diseases, Singapore |
| Oon Tek Ng | MPH | National Centre for Infectious Diseases, Singapore |
| Monica Chan | BMBS | National Centre for Infectious Diseases, Singapore |
| Kalisvar Marimuthu | MBBS | National Centre for Infectious Diseases, Singapore |
| Shawn Vasoo | MBBS | National Centre for Infectious Diseases, Singapore |
| Chen Seong Wong | MBBS | National Centre for Infectious Diseases, Singapore |
| Tau Hong Lee | MBBS | National Centre for Infectious Diseases, Singapore |
| Sapna Pradip Sadarangani | MBBS | National Centre for Infectious Diseases, Singapore |
| Ray Junhao Lin | MMed | National Centre for Infectious Diseases, Singapore |
| Mucheli Sharavan Sadasiv | MMed | National Centre for Infectious Diseases, Singapore |
| Deborah Hee Ling Ng | MBChB | National Centre for Infectious Diseases, Singapore |
| Chiaw Yee Choy | MBBS | National Centre for Infectious Diseases, Singapore |
| Glorijoy Shi En Tan | MBBS | National Centre for Infectious Diseases, Singapore |
| Yu Kit Tan | MBBS | National Centre for Infectious Diseases, Singapore |
| Sean Wei Xiang Ong | MBBS | National Centre for Infectious Diseases, Singapore |
| Stephanie Sutjipto | MBBS | National Centre for Infectious Diseases, Singapore |
| Pei Hua Lee | MBChB | National Centre for Infectious Diseases, Singapore |
| Jun Yang Tay | MBBS | National Centre for Infectious Diseases, Singapore |
| Ding Ying | PhD | National Centre for Infectious Diseases, Singapore |
| Bo Yan Khoo | MBBS | National Centre for Infectious Diseases, Singapore |
| Woo Chiao Tay | MBBS | National Centre for Infectious Diseases, Singapore |
| Gabrielle Ng | MBBS | National Centre for Infectious Diseases, Singapore |
| Yun Yuan Mah | MBBS | National Centre for Infectious Diseases, Singapore |
| Wilnard Tan | MBBS | National Centre for Infectious Diseases, Singapore |
| Sennen Jin Wen Lew | MBBS | Tan Tock Seng Hospital, Singapore |
| Raymond Kok Choon Fong | MBBS | Changi General Hospital, Singapore |
| Helen May Lin Oh | MMed | Changi General Hospital, Singapore |
| Jaime Mei Fong Chien | MMed | Changi General Hospital, Singapore |
| Humaira Shafi | MBBS | Changi General Hospital, Singapore |
| Hau Yiang Cheong | MBBS | Changi General Hospital, Singapore |
| Darren Cheng Han Teo | MBBS | Changi General Hospital, Singapore |
| Thuan Tong Tan | PhD | Singapore General Hospital, Singapore |
| Ban Hock Tan | MBBS | Singapore General Hospital, Singapore |
| Jenny Guek Hong Low | MPH | Singapore General Hospital, Singapore |
| Limin Wijaya | MBBS | Singapore General Hospital, Singapore |
| Indumathi Venkatachalam | MPH | Singapore General Hospital, Singapore |
| Ying Ying Chua | MMed | Singapore General Hospital, Singapore |
| Benjamin Pei Zhi Cherng | MBBS | Singapore General Hospital, Singapore |
| Yvonne Fu Zi Chan | MMed | Singapore General Hospital, Singapore |
| Ghee Chee Phua | MBBS | Singapore General Hospital, Singapore |
| Ken Junyang Goh | MD | Singapore General Hospital, Singapore |
| Jade Xiao Jue Soh | MBBS | Sengkang General Hospital, Singapore |
| Shuwei Zheng | MBBS | Sengkang General Hospital, Singapore |
| Pushpalatha Bangalore Lingegowda | MBBS | Sengkang General Hospital, Singapore |
| Wee Ming Peh | MBBS | Sengkang General Hospital, Singapore |
| Yi Lin Lee | MBBS | Sengkang General Hospital, Singapore |
| Jun Yang Ho | MBChB | Sengkang General Hospital, Singapore |
| April Yu Jie Chia | MBBS | Sengkang General Hospital, Singapore |
| Li Lin | MD | Ng Teng Fong General Hospital, Singapore |
| Say Tat Ooi | MD | Khoo Teck Puat Hospital, Singapore |
| Tambyah Paul Anantharajah | MBBS | National University Hospital, Singapore |
| Jyoti Somani | MD | National University Hospital, Singapore |
| Jolene Ee Ling Oon | MB BCh | National University Hospital, Singapore |
| Gabriel Zherong Yan | MBBS | National University Hospital, Singapore |
