# Supplementary material for: Clinical features and predictors of severity in COVID-19 patients with critical illness in Singapore
Source: Sci Rep. 2021 Apr 5;11:7477. doi: 10.1038/s41598-021-81377-3 (PMC8021583; doi:10.1038/s41598-021-81377-3)
Supplement: Supplementary file 2 — Supplementary Information 2. [file 41598_2021_81377_MOESM2_ESM.doc]

**Online Data Supplement**

Title: **Clinical Features and Predictors of Severity in COVID-19 Patients with Critical Illness in Singapore**

Ser Hon Puah, MBBS^1^, Barnaby Edward Young, MBBchir^1,2,9^, Po Ying Chia, MBBS^1,2,9^, Vui Kian Ho, MBBS^3^, Jiashen Loh, MBBS^3^, Roshni Sadashiv Gokhale, MBBS^4^, Seow Yen Tan, MBBS^5^, Duu Wen Sewa, MBBS^5^, Shirin Kalimuddin, MBBS^5,10^, Chee Keat Tan, MD^8^, Surinder KMS Pada, MBBS^8,11^, Matthew Edward Cove, MBChB^6,11^, Louis Yi Ann Chai, MBBS^6,11^, Purnima Parthasarathy, MBBS^7^, Benjamin Choon Heng Ho, MBBS^1^, Jensen Jiansheng Ng, MBBS^1^, Li Min Ling, MBBS^1,2,9^, John A Abisheganaden, MBBS^1,9^, Vernon JM Lee, MBBS^12,13^, Cher Heng Tan, MBBS^1,9^, Raymond TP Lin, MBBS^2,11^, Yee Sin Leo, MBBS^1,2,9,11,13^, David C Lye, MBBS^1,2,9,11^, Tsin Wen Yeo, MBBS^1,2,9,14^ on behalf of Singapore 2019 novel coronavirus outbreak research team.

^1^Tan Tock Seng Hospital, Singapore,^2^National Centre for Infectious Diseases, Singapore, ^3^Sengkang General Hospital, Singapore, ^4^Changi General Hospital, Singapore, ^5^Singapore General Hospital, Singapore, ^6^National University Hospital, Singapore, ^7^Khoo Teck Puat Hospital, Singapore, ^8^Ng Teng Fong General Hospital, Singapore, ^9^Lee Kong Chian School of Medicine, Singapore, ^10^Duke-NUS Medical School, Singapore, ^11^Yong Loo Lin School of Medicine, Singapore, ^12^Ministry of Health, Singapore, ^13^Saw Swee Hock School of Public Health, Singapore, ^14^Menzies School of Health Research, Charles Darwin University, Darwin, Northern Territory, Australia

**Supplementary Tables and Figures**

**Table E1: Area under curve (AUC) estimates for receiver operator characteristic (ROC) curves**

Receiver operator curves (ROC) for baseline haematological and biochemical factors at predicting need for intubation.

58 patients included, 8 who required intubation and 50 controls. Two individuals excluded who required intubation at presentation.

|  | **AUC** | **Criterion*** | **Sensitivity** | **Specificity** | **p-value** |
| --- | --- | --- | --- | --- | --- |
| Lymphocyte count (x 10^9^/L) | 0.883 (0.771 to 0.952) | ≤0.68 (≤0.48 to ≤1.11) | 75% (34.9 to 96.8) | 94% (83.5 to 98.7) | <0.001 |
| Neutrophil count (x 10^9^/L) | 0.778 (0.649 to 0.876) | >4.61 (>3.02 to >6.05) | 50% (15.7 to 84.3) | 98% (89.4 to 99.9) | 0.01 |
| Neutrophil:lymphocyte ratio (NLR) | 0.885 (0.774 to 0.954) | >4.42 (>1.18 to >5.66) | 87.5% (47.3 to 99.7) | 88.0% (75.7 to 95.5) | <0.001 |
| CRP, mg/L | 0.932 (0.816 to 0.86) | >68.7 (>36.7 to >97.5) | 87.5% (47.3 to 99.7) | 89.2 (74.6 to 97) | <0.001 |
| LDH, U/L | 0.803 (0.664 to 0.903) | >796 (>458 to >996) | 57.1% (18.4 to 90.1) | 97.6% (87.4 to 99.9) | 0.002 |

CRP = C-reactive protein; LDH = lactate dehydrogenase.

*Optimum cut-off point for sensitivity and specificity estimated with Youden Index and bootstrap confidence intervals (1000 iterations).

**Table E2: Baseline Characteristics and Laboratory Results of Mechanically Ventilated COVID-19 Patients on Day of ICU Admission**

| **Patient Number** | **Age**  **(years)** | **Gender** | **Co-Morbidities** | **Onset of Symptoms to Intubation (days)** | **Time from Hospital admission to Intubation**  **(days)** | **White Blood Cells**  **(x10^9^/L)** | **Lymphocyte**  **Count**  **((x10^9^/L)** | **Lactate**  **Dehydrogenase**  **(U/L)** | **Creatinine**  **(µmol/L)** | **C-Reactive Protein**  **(mg/L)** |
| --- | --- | --- | --- | --- | --- | --- | --- | --- | --- | --- |
| 1 | 47 | Female | Nil | 7 | 6 | 6.8 | 0.52 | 650 | 55 | 190.5 |
| 2 | 52 | Male | Diabetes Mellitus,  Fatty liver, Obesity | 9 | 1 | 5.1 | 0.5 | 646 | 81 | 56 |
| 3 | 39 | Male | Nil | 6 | 1 | 8.2 | 0.76 | 1908 | 73 | 202.2 |
| 4 | 71 | Male | Diabetes Mellitus | 9 | 3 | 6.2 | 0.61 | 632 | 62 | 248.7 |
| 5 | 62 | Male | Gastroesophageal  Reflux | 8 | 0 | 9.6 | 0.49 | 1460 | 93 | 112.9 |
| 6 | 36 | Male | Nil | 6 | 0 | 2.3 | 0.25 | 396 | 76 | 291.1 |
| 7 | 39 | Female | Nil | 9 | 4 | 5.91 | 0.34 | Not available | 67 | 178 |
| 8 | 54 | Male | Hypertension, Hyperlipidaemia | 8 | 1 | 6.87 | 0.55 | 402 | 110 | 115 |
| 9 | 64 | Male | Nil | 9 | 3 | 11.6 | Not available | 896 | 78 | 140 |
| 10 | 53 | Male | Diabetes Mellitus without chronic complications,  Obesity | 10 | 1 | 10.4 | 0.4 | Not available | 67 | 199.6 |

**Table E3: Severity of Illness, Ventilator Data and Arterial Blood Gas Results of Mechanically Ventilated COVID-19 Patients on Day of Intubation**

| Patient Number | APACHE II | SOFA | pH before intubation | PaO_2_  (mmHg) before intubation | PaCO_2_  (mmHg)  before intubation | PaO_2_/Fio_2_  ratio before intubation | PaO_2_/FiO_2_ ratio after intubation | Plateau  pressure  (first 24 hours) | FiO2  (first 24 hours) | PEEP at the end of 24 hours | Paralysed  Yes/No | Prone positoning  Yes/NO | Duration of Ventilation  (Days) |
| --- | --- | --- | --- | --- | --- | --- | --- | --- | --- | --- | --- | --- | --- |
| 1 | 19 | 6 | 7.44 | 94 | 36 | 117 | 332.5 | 18 | 0.6 | 8 | No | No | 3 |
| 2 | 22 | 10 | 7.33 | 69 | 42 | 138 | 187 | 18 | 0.5 | 13 | No | No | 7 |
| 3 | 24 | 12 | 7.30 | 76 | 37 | 76 | 107 | 25 | 1.0 | 16 | Yes | Yes | Currently Still Ventilated |
| 4 | 20 | 12 | 7.47 | 62 | 33 | 124 | 148 | 19 | 0.6 | 10 | No | No | 17 |
| 5 | 18 | 7 | 7.38 | 90 | 36 | 90 | 230 | 20 | 1.0 | 10 | No | No | 5 |
| 6 | 23 | 10 | 7.38 | 112 | 32 | 140 | 217 | 26 | 0.8 | 14 | Yes | Yes | 9 |
| 7 | 16 | 4 | 7.40 | 89.3 | 38.1 | 89.3 | 161 | 28 | 1.0 | 14 | Yes | No | 6 |
| 8 | 20 | 9 | 7.46 | 48.9 | 30 | 48.9 | 70 | Not available | 1.0 | 14 | Yes | No | 17 |
| 9 | 17 | 2 | 7.31 | 63 | 45 | 90 | 134 | 24 | 0.7 | 10 | Yes | No | Currently Still Ventilated |
| 10 | 16 | 5 | 7.42 | 51.4 | 37 | 128.5 | 174.6 | NA | 0.8 | 12 | No | No | 5 |

ARDS = acute respiratory distress syndrome; SOFA = Sequential Organ Failure Assessment; PaO_2_ = Partial pressure of oxygen; PaCO_2_ = partial pressure of carbon dioxide; FiO_2_ = Fraction of inspired oxygen; PEEP = Positive end expiratory pressure

**Table E4: Complications and Treatment of Mechanically Ventilated Patients with COVID-19**

| **Patient Number** | **ARDS** | **Acute**  **Respiratory Injury** | **Shock** | **AKI** | **Nosocomial**  **Infections** | **COVID-19 Targeted Therapy** | **Antivirals** | **Antibiotics** | **CRRT** | **Duration of ICU Stay**  **(Days)** |
| --- | --- | --- | --- | --- | --- | --- | --- | --- | --- | --- |
| 1 | No | Yes | No | No | No | Lopinavir/Ritonavir | Oseltamivir  (2 doses) | None during ICU stay | No | 5 |
| 2 | Yes | No | No | No | No | Lopinavir/Ritonavir | Oseltamivir  (2 doses) | Piperacillin/Tazobactam 5 days  Meropenem 2 days | No | 9 |
| 3 | Yes | No | Yes | Yes | Yes | Lopinavir/Ritonavir  Interferon-beta | Oseltamivir  (2 doses) | Clarithromycin 1 day  Meropenem 11 days  Vancomycin 4 days  Anidulafungin 7 days | Yes | 20 (still intubated) |
| 4 | Yes | No | No | No | No | Lopinavir/Ritonavir  Interferon-beta | No | Meropenem 7 days | No | 18 |
| 5 | Yes | No | No | No | No | Lopinavir/Ritonavir | Oseltamivir  (1 dose) | Benzyl Penicillin 2 days  Azithromycin 2 days  Ceftazidime 2 days | No | 6 |
| 6 | Yes | No | Yes | No | Yes | Lopinavir/Ritonavir  Interferon-beta | No | Meropenem 5 days  Amoxicillin/Clavulanate 3 days  Piperacillin/Tazobactam 2 days | No | 10 |
| 7 | Yes | No | Yes | No | No | Lopinavir/Ritonavir | No | Ceftriaxone 2 days  Clarithromycin 2 days | No | 7 |
| 8 | Yes | No | No | No | Yes | Lopinavir/Ritonavir | No | Azithromycin 4 days  Meropenem 4 days  Piperacillin/Tazobactam 7 days  Vancomycin 7 days  Meropenem 2 day  Ertapenem 5 days | No | 19 |
| 9 | Yes | No | No | No | Yes | Lopinavir/Ritonavir | No | Meropenem 3 days  Ceftriaxone 6 days  Piperacillin/Tazobactam 2 days  Vancomycin 5 days | No | Extubated after 11 days  Reintubated after 6 days off ventilator |
| 10 | Yes | No | No | No | No | Lopinavir/Ritonavir | No | Ceftriaxone 2 days  Azithromycin 2 days | No | 6 |

ARDS = acute respiratory distress syndrome; AKI = acute kidney injury; CRRT = continuous renal replacement therapy

**Figure 1E**

Plot of Receiver operator characteristic (ROC) curves with area under curve (AUC) estimates:

1. Lymphocyte count
2. Neutrophil count
3. NLR = Neutrophil:lymphocyte ratio
4. C-Reactive Protein
5. LDH = Lactate dehydrogenase
